# Effects of common groundwater ions on chromate removal by magnetite: importance of chromate adsorption

**DOI:** 10.1186/s12932-016-0033-9

**Published:** 2016-04-29

**Authors:** Amanda H. Meena, Yuji Arai

**Affiliations:** School of Agricultural, Forest and Environmental Sciences, Clemson University, Clemson, SC 29634 USA; Department of Natural Resources and Environmental Sciences, University of Illinois at Urbana-Champaign, Urbana, IL 61801 USA

**Keywords:** Chromate, Cr, Groundwater Ions, Magnetite, Adsorption, Surface Speciation, Reduction, Sulfate, Nitrate, X-ray absorption spectroscopy

## Abstract

**Background:**

Reductive precipitation of hexavalent chromium (Cr(VI)) with magnetite is a well-known Cr(VI) remediation method to improve water quality. The rapid (<a few hr) reduction of soluble Cr(VI) to insoluble Cr(III) species by Fe(II) in magnetite has been the primary focus of the Cr(VI) removal process in the past. However, the contribution of simultaneous Cr(VI) adsorption processes in aged magnetite has been largely ignored, leaving uncertainties in evaluating the application of in situ Cr remediation technologies for aqueous systems. In this study, effects of common groundwater ions (i.e., nitrate and sulfate) on Cr(VI) sorption to magnetite were investigated using batch geochemical experiments in conjunction with X-ray absorption spectroscopy.

**Results:**

In both nitrate and sulfate electrolytes, batch sorption experiments showed that Cr(VI) sorption decreases with increasing pH from 4 to 8. In this pH range, Cr(VI) sorption decreased with increasing ionic strength of sulfate from 0.01 to 0.1 M whereas nitrate concentrations did not alter the Cr(VI) sorption behavior. This indicates the background electrolyte specific Cr(VI) sorption process in magnetite. Under the same ionic strength, Cr(VI) removal in sulfate containing solutions was greater than that in nitrate solutions. This is because the oxidation of Fe(II) by nitrate is more thermodynamically favorable than by sulfate, leaving less reduction capacity of magnetite to reduce Cr(VI) in the nitrate media. X-ray absorption spectroscopy analysis supports the macroscopic evidence that more than 75 % of total Cr on the magnetite surfaces was adsorbed Cr(VI) species after 48 h.

**Conclusion:**

This experimental geochemical study showed that the adsorption process of Cr(VI) anions was as important as the reductive precipitation of Cr(III) in describing the removal of Cr(VI) by magnetite, and these interfacial adsorption processes could be impacted by common groundwater ions like sulfate and nitrate. The results of this study highlight new information about the large quantity of adsorbed Cr(VI) surface complexes at the magnetite-water interface. It has implications for predicting the long-term stability of Cr at the magnetite-water interface.

**Graphical abstract:**
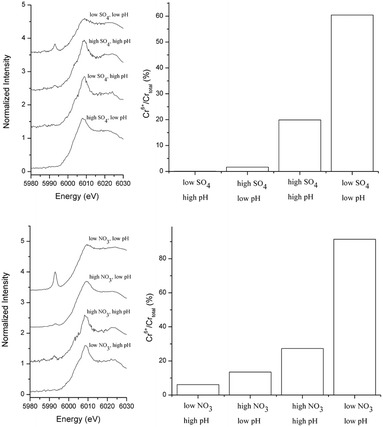
Effects of background anions (sulfate and nitrate) on the Cr(VI) surface coverage at the magnetite-water interface at pH 4 and 9

## Background

Hexavalent chromium [Cr(VI) or chromate], has been recognized as one of the major toxic substances by the United States Environmental Protection Agency (USEPA) [1] because of its carcinogenic effects [2]. In a recent report by Environmental Working Group, [Cr(VI)] in tap water tested from 25 out of 35 American cities was greater than a proposed limit of 0.06 µg L^−1^ by the state of California EPA [3]. While the occurrence of Cr in these water resources is contributed by anthropogenic (e.g., steel mills, leather-tanning facilities) and indigenous sources (weathering of rocks and soils), there is an imminent interest to reduce the concentration of Cr from drinking water resources.

The reduction of Cr(VI) by synthetic magnetite has been frequently studied for the remediation of Cr(VI) contaminated waters in the past [4–7]. In a Cr(VI) tetrahedral ion, t_2g_ and e.g. orbitals are empty. It accepts three electrons from the t_2g_ (pi) orbital of three ferrous ions, filling half of the t_2g_ orbital, Cr(III). It is at the ground-state electron configuration in an octahedral environment. It is well documented that Fe(II) in magnetite facilitates the reduction of Cr(VI) to Cr(III), subsequently forming Cr(III) hydroxide and or a mixed Cr(III)-Fe(III) hydroxide surface precipitate at the surface of an iron oxide [8–11]. These reductive precipitation reactions often refer to Cr(VI) sorption to magnetite. It is important to note that the term, sorption, was used to describe both precipitation and adsorption reactions on the mineral surfaces throughout the text. The Cr(VI) sorption reaction in magnetite is pH dependent. Sorption increases with decreasing pH [4, 6, 12]. Based on the following half-reactions 1–3 [13, 14], overall reactions of Cr(VI) reduction by Fe(II) can be written in reactions 4 and 5.

1 $${\text{CrO}}_{4}^{2 - } + {\text{ 8H}}^{ + } + {\text{ 3e}}^{ - } \longrightarrow {\text{ Cr}}^{ 3+ } + {\text{ 4H}}_{ 2} {\text{O}} \hspace {10 mm} {E}^{\text{o}} ( {\text{V) }} = \, - 0. 1 1 {\text{ volts}}$$

2 $$3 {\text{Fe}}^{2 + } + 4 {\text{H}}_{ 2} {\text{O}} \longrightarrow {\text{Fe}}_{ 3} {\text{O}}_{ 4} + 8 {\text{H}}^{ + } + 2 {\text{e}}^{ - } \hspace {10 mm} {\text{E}}^{\text{o}} ( {\text{V) }} = { 1}.0 8 {\text{ volts}}$$

3 $${\text{Fe}}^{ 2+ } \longrightarrow {\text{ Fe}}^{ 3+ } + {\text{ e}}^{ - } \hspace {10 mm} {\text{E}}^{\text{o}} = - 0. 7 7 7 {\text{ volts}}$$

4 $$2 {\text{CrO}}_{4}^{2 } + 9 {\text{Fe}}^{ 2+} + 4 {\text{H}}_{ 2} {\text{O}} \longrightarrow 2 {\text{Cr}}^{ 3+} + {\text{ 3Fe}}_{ 3} {\text{O}}_{ 4} + 8 {\text{H}}^{+}$$

5 $${\text{CrO}}_{4}^{2-} + 3 {\text{Fe}}^{2+} + {\text{ 8H}}^{+} \longrightarrow {\text{Cr}}^{3+} + {\text{ 3Fe}}^{3+} + 4 {\text{H}}_{ 2} {\text{O}}$$

Kendelewicz and other researchers postulated a two-step mechanism to explain the Cr(VI) sorption process: electrostatic attraction of Cr(VI) anions, followed by the electron transfer reaction between Cr(VI) and the structural Fe(II) to form Cr(III)(OH)_3_ [8, 12, 15, 16]. The Cr(VI) reduction mechanism was accompanied by simultaneous homogenous oxidation of Fe(II) released by passivation of magnetite [7]. Especially at basic conditions, Fe(II) in magnetite is highly susceptible to auto-oxidation, resulting in a decrease in Cr(VI) reduction [12]. Although the above sorption mechanisms were suggested, it is poorly understood how these steps are interfered by common ions in natural and waste waters. Our water resources usually contain ions like nitrate and sulfate that could potentially interfere with the formation of Cr(VI) precursor complex on the magnetite surface and or electron transfer reactions. This could potentially result in ineffective Cr(VI) immobilization with magnetite. Nitrate concentration in surface water ranges from 0.1 to 20 mg L^−1^, and it can be as high as 30 mg L^−1^ in wastewater [17]. Sulfate in domestic sewage effluents can be as high as 500 mg L^−1^, and up to several thousand mg L^−1^ in some industrial effluents [18]. It is possible that these anions can potentially interfere with Cr(VI) immobilization by magnetite via (1) competitive adsorption of nitrate and sulfate and (2) competitive electron transfer reactions.

In general, the strength of oxyanion complexation on metal oxyhydroxide surfaces can be predicted using the shared charge value (SCV), which is the positive oxyanion charge divided by the number of bonded O atoms. The lower the SCV, the stronger the affinity of oxyanion sorption. The SCV for NO_3_^−^ is 1.67 while SO_4_^2−^ and CrO_4_^2−^ both have a SCV of 1.5, indicating that the latter oxyanions have a slightly stronger metal-oxyanion ionic bond. Based on the SCV, one can expect that nitrate will not strongly perturb the initial CrO_4_^2−^ adsorption step. However, sulfate could compete for sorption sites, resulting in less chromate sorption and or suppression of electron transfer reactions.

Competitive Fe(II) redox reactions by nitrate could also influence Cr(VI) reduction. Based on the standard state redox potential of half-reactions [13] and $\varDelta {\text{G}}^{\text{o}} = \, - {\text{n}}F\left( {{\text{E}}_{\text{ox}}^{\text{o}} + {\text{ E}}_{\text{red}}^{\text{o}} } \right)$, where n is the number of moles of e^−^ from balanced redox reaction and *F* is the Faraday constant (96,487 J V^−1^ mol ^−1^), it is clear that the thermodynamic favorability of the reduction of Cr(VI) by Fe(II) is most preferred over the reduction of nitrate/sulfate under the equilibrium condition. However, if Cr(VI) co-exists with nitrate, Fe(II) in magnetite, as surface bound/crystal defects, could potentially be depleted by the reduction of nitrate, resulting in less Cr(VI) reduction. Kinetically controlled effects cannot be excluded.

6 $${\text{Cr}}_{ 2} {\text{O}}_{7}^{ 2- } + {\text{ 14H}}^{ + } + {\text{ 6e}}^{ - } \longrightarrow 2 {\text{Cr}}^{ 3+ } + {\text{ 7H}}_{ 2} {\text{O}} \hspace{10 mm}{E}^{\text{o}} = \, + 1. 3 6 {\text{ volts}}$$

7 $${\text{NO}}_{3}^{ - } + { 1}0{\text{H}}^{ + } + {\text{ 8e}}^{ - } \longrightarrow {\text{ NH}}_{4}^{ + } + {\text{ 3H}}_{ 2} {\text{O}} \hspace{10 mm}{E}^{\text{o}} = + 0. 8 8 2 {\text{ volts}}$$

8 $${\text{NO}}_{3}^{{^{ - } }} + {\text{ 2H}}^{ + } + {\text{ 2e}}^{ - } \longrightarrow {\text{ NO}}_{2}^{ - } + {\text{ H}}_{ 2} {\text{O}} \hspace{10 mm}{E}^{\text{o}} = + 0. 8 3 7 {\text{ volts}}$$

9 $${\text{SO}}_{ 4}^{ 2- } + {\text{ 9H}}^{ + } + {\text{ 8e}}^{ - } \longrightarrow {\text{ HS}}^{ - } + {\text{ 4H}}_{ 2} {\text{O}} \hspace {10 mm} {E}^{\text{o}} = + 0. 2 4 8 {\text{ volts}}$$

The objective of this study was to investigate the effects of nitrate and sulfate on Cr(VI) removal by magnetite, as a function of pH and ionic strength (0.01 vs. 0.1) through batch sorption experiments. As reviewed above, numerous investigations in the same system [i.e., Cr(VI) in magnetite] were dedicated to the characterization of reaction products in corroded magnetite. This study, instead, focuses on the macroscopic behavior of the Cr(VI) removal process by magnetite in two different electrolyte systems. To understand the effect of these electrolytes on the Cr surface species, in situ Cr K-edge X-ray absorption spectroscopy (XAS) measurements were also conducted. These analyses allow for a greater understanding of the Cr(VI) removal capacity of magnetite in natural water systems.

## Results and discussion

### PZSE of magnetite

The PZSE of magnetite used in this study was determined using batch titrations with 0.01, 0.05 and 0.1 M NaNO_3_, and was approximately 5.63, slightly lower than reported PZSE and PZC values of synthetic magnetite, 6.3–6.8 [12, 19, 20]. Salazar-Camacho and co-workers also reported the isoelectric point (IEP) of two nano-magnetite samples to be 6.2 for <5 µm and 6.7 for <50 nm particles [21]. This discrepancy may be due to surface oxidation and or impurities [22–24]. In the literature, comparatively lower PZSE values have been reported with natural magnetite samples [21, 25, 26].

### Pseudo-equilibrium sorption experiments

**Fig. 1:**
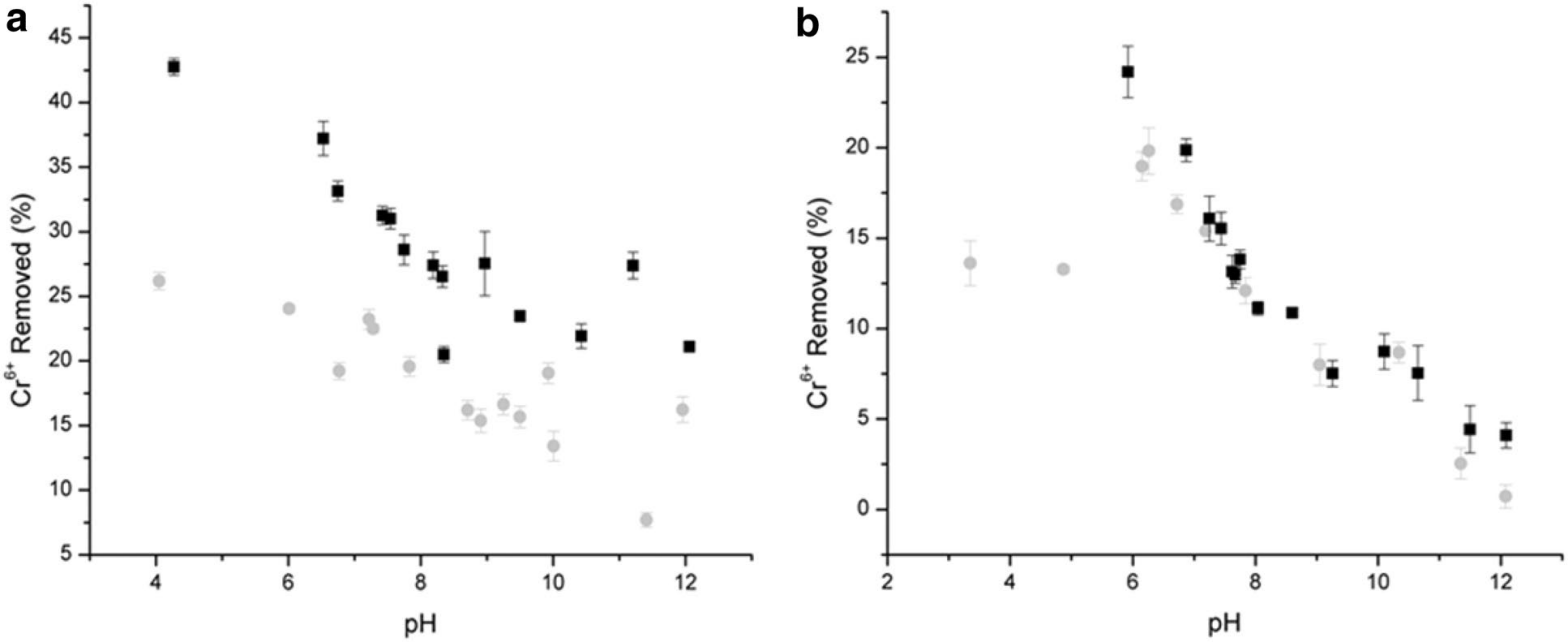
Chromate sorption envelopes in magnetite after 24 h in the following background electrolyte solutions **a** Na_2_SO_4_ **b** NaNO_3_. *Black squares* and *grey circles* represent low (0.0016 M Na_2_SO_4_, 0.01 M NaNO_3_) and high (0.016 M Na_2_SO_4_, 0.1 M NaNO_3_) ionic strengths, respectively

The results of CrO_4_^2−^ sorption envelope experiments in magnetite are shown in Fig. 1. In the following sections, macroscopic behavior of chromate sorption to magnetite is discussed in terms of pH, ionic strength, and type of electrolytes (NaNO_3_ and Na_2_SO_4_).

### Effects of pH

In both electrolytes, chromate sorption is pH dependent. The sorption generally increases with decreasing pH from 10 to 4 although a few data points at pH <5 (Fig. 1b) are influenced by the dissolution of solids. Similar pH dependent chromate sorption behavior has been documented in several studies [4–6, 12, 27, 28]. Assuming that Cr(VI) is present, the sorption trend can be explained by the aqueous speciation of chromate and the surface charge density of magnetite at given pH values.

**Fig. 2:**
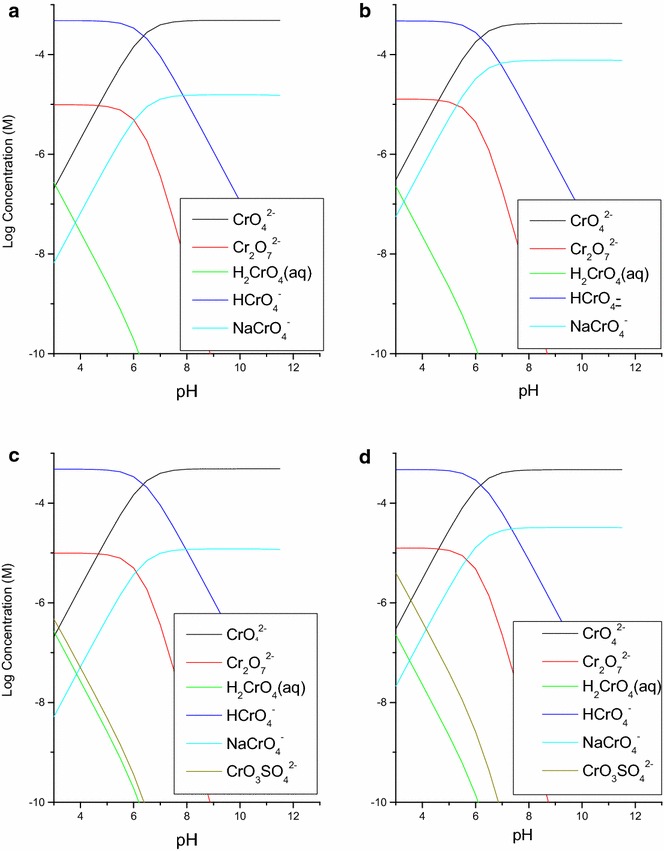
Aqueous speciation diagrams of 0.5 mM Cr(VI)O_4_^2−^ over pH 3–12 using Visual MINTEQ in the following background media **a** and **b** 0.01 and 0.1 M NaNO_3_, respectively. **c** and **d** 0.0016 and 0.016 M Na_2_SO_4_, respectively

Aqueous speciation of chromate under all conditions was calculated using Visual MINTEQ version 3.0 [29]. In both electrolytes, negatively charged chromate species are generally observed (Fig. 2). The HCrO_4_^−^ species is dominant at approximately pH <6 and CrO_4_^2−^ at pH >6 with a minor contribution from NaCrO_4_^−^ species. The dissociation constant of hydrogen chromate (3.1 × 10^−7^, pKa 6.51) agrees with the predominance of CrO_4_^2−^ as the major aqueous species at experimental pH values 4–10 [30].

At pH <5 (below PZSE of magnetite), surfaces of magnetite are positively charged. Chromate anions are expected to be strongly attracted to the surfaces via electrostatic interactions. This supports a 1st step to form the precursor complex prior to the electron transfer reaction suggested by Kendelewicz and co-workers [8, 15].

At pH >5, more negatively charged surfaces will be developed, further reducing the attraction of anions, like chromate. However, some sorption occurred at pH 8–11, possibly suggesting inner-sphere sorption mechanisms via ligand exchange reactions.

### Effects of ionic strength

Although there are some variable data points in each electrolyte experiment (Fig. 1a, b), one can clearly see two distinct ionic strength effects on CrO_4_^2−^ sorption. In the sulfate media, Cr(VI) uptake was affected by changes in ionic strength. The sorption drastically decreased when ionic strength was increased from 0.0016 to 0.016 M sulfate at pH 3–12. Average Cr(VI) removal is 10.4 % lower at higher sulfate concentration. White and Peterson also previously reported the effects of SO_4_^2−^ concentrations (0.01–0.1 M) on chromate sorption [7]. On the other hand, little effect of ionic strength effect was observed in the nitrate media. This observation is consistent with other studies that found negligible effects of NO_3_^−^ concentration on Cr(VI) removal by magnetite [31].

Hayes and co-workers previously proposed an indirect macroscopic method for distinguishing inner-sphere from outer-sphere complexes by examining ionic strength effects of inert electrolytes on oxyanion sorption envelopes coupled with the generalized triple layer model [32]. Accordingly, inner-sphere complexes are not greatly affected by ionic strength, whereas the presence of outer-sphere complexes is indicated by a shift in the pH with changing ionic strength due to competitive sorption with counter anions. Based on the theory, one can suggest that chromate predominantly forms inner-sphere complexes in the NaNO_3_ media at pH 4–11, whereas chromate could adsorb to magnetite as a mixture of inner- and outer-sphere complexes in the Na_2_SO_4_ media. In the high ionic strength of Na_2_SO_4_, it is possible that the surface speciation of chromate is predominantly inner-sphere complexes at pH 6–12. These interpretations of sorption mechanisms, however, are contradicted in two different background electrolyte media, which is likely attributed to differences in inertness between nitrate and sulfate ions and or redox reaction at the surfaces. The macroscopic observation is useful in evaluating chromate removal from aqueous solution under different reaction conditions. However, the removal of Cr(VI) from solution should not be interpreted as the chemi-sorption of Cr(VI) anions in magnetite without any spectroscopic evidence. In the XAS analysis section below, chemical speciation of Cr on magnetite surfaces is discussed.

### Effects of sulfate and nitrate

When the total Cr retention was compared in these electrolyte systems (Fig. 1), the sulfate system yielded more Cr retention by magnetite. Based on the SCV argument discussed above, sulfate should have interfered with the adsorption of CrO_4_^2−^. However, this is not the case. The nitrate system yielded less Cr retention. It is likely that a different factor was involved in the reactions (Fig. 1). As reported by several previous studies [4–7], Cr(VI) uptake by magnetite is attributed to the reduction of Cr(VI) by Fe(II) in magnetite. If the surface bound Fe(II) and the Fe(II) in crystal defects are consumed by other anions like nitrate, the presence of nitrate should lower the reduction of Cr(VI), resulting in less Cr uptake by magnetite. When ΔG^o^ of oxyanion reduction was estimated using the half reaction Eqs. 3 and 6–9, Cr(VI) reduction is most favorable (ΔG^o^ = −56.25 kJ), followed by nitrate reduction to nitrite (ΔG^o^ = −11.57 kJ), nitrate reduction to ammonium (ΔG^o^ = −10.13 kJ), and sulfate reduction to bisulfide (ΔG^o^ = 385.17 kJ). This clearly suggests that sulfate reduction does not favorably occur at the standard state. However, nitrate could competitively oxidize surface available Fe(II) in magnetite. Although kinetics of competitive chromate and nitrate reduction was not measured in this research, it is clear that nitrate was more competitively oxidizing Fe(II) than sulfate, possibly resulting in less Cr retention in the nitrate system.

### XAS analysis

To better assess the macroscopic observation discussed above, the chemical speciation of Cr on the magnetite surface was investigated using XAS. Effects of ionic strength, pH and kinetics on the Cr surface speciation are discussed below.

### Effect of ionic strength on Cr surface speciation

**Fig. 3:**
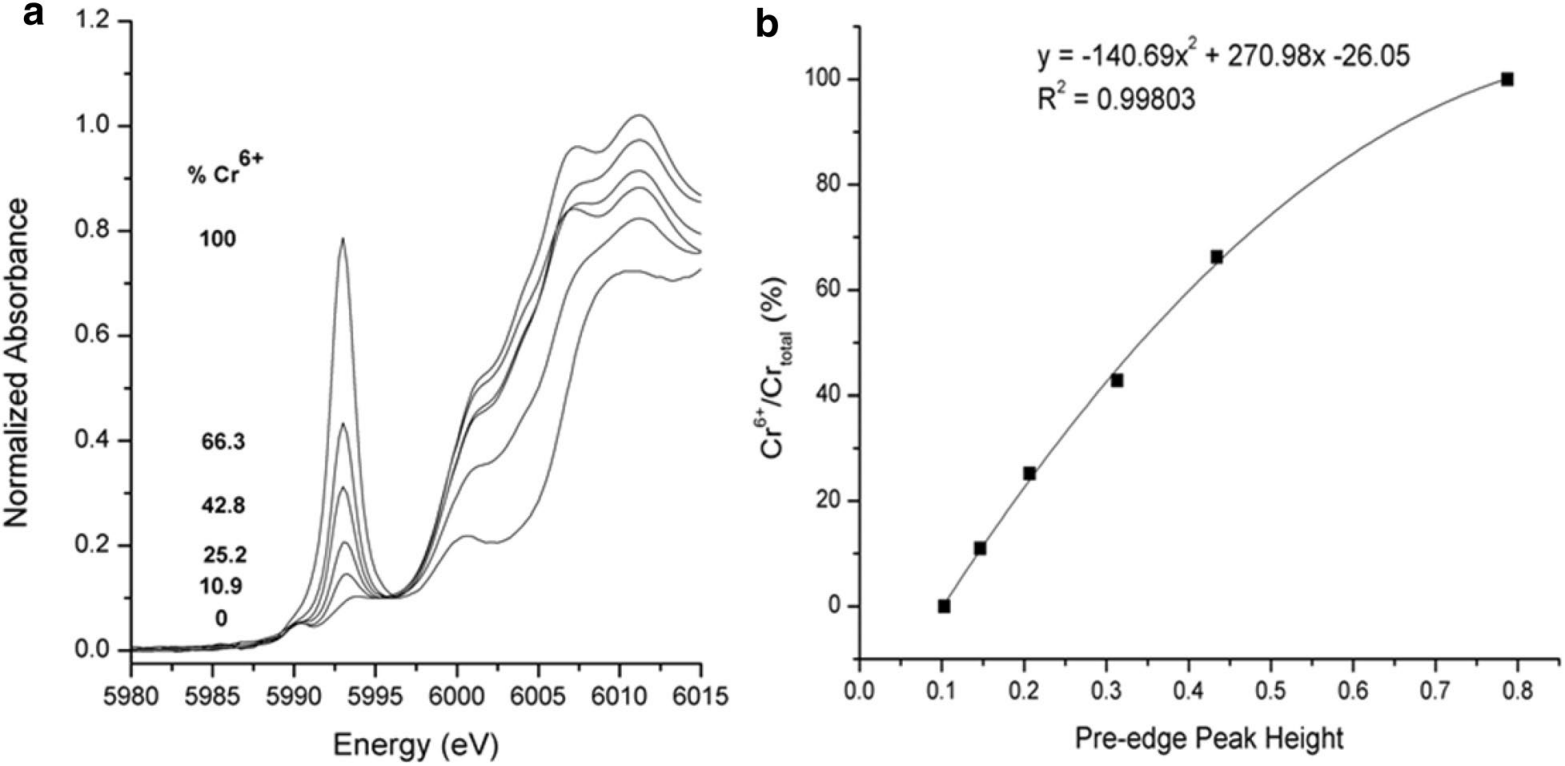
**a** Pre-edge features of bulk XANES spectra of reference salt mixtures (K_2_Cr(VI)O_4_ and Cr_2_(III)O_3_) **b** The intensity of Cr(VI) pre-edge height of reference spectra in Fig. 3a as a function of Cr(VI)/Total Cr (%)

**Fig. 4:**
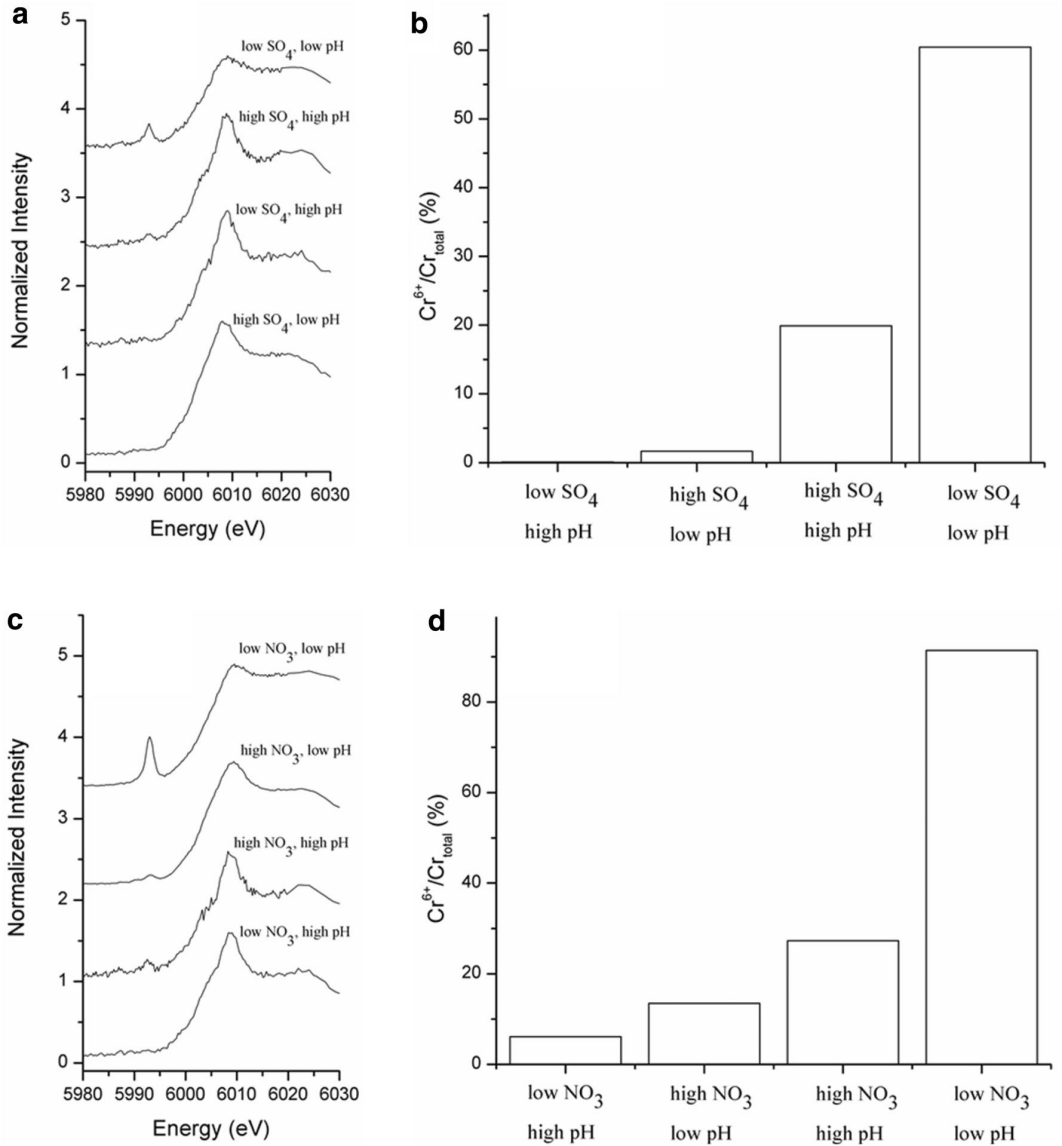
Chromium surface speciation at the magnetite-water interface as a function of pH (4.02 ± 0.1 and 9.04 ± 0.03) and electrolyte concentrations. High and low pH values correspond to pH 9 and 4, respectively. **a** Pre-edge features of Cr XANES spectra from Cr(VI) reacted magnetite under 0.0016 M (low SO_4_) and 0.016 M (low SO_4_) Na_2_SO_4_. **b**  % Cr(VI) fraction in the sorption samples shown in Fig. 4a. **c** Pre-edge features of Cr K-edge XANES spectra from Cr(VI) reacted magnetite under 0.01 M (low NO_3_) and 0.1 M (high NO_3_) NaNO_3_. **d** Data assessed by XANES pre-edge analysis shown in Fig. 4c. The fraction of  % Cr(VI) was estimated using the Cr(VI)standard curve in Fig. 3

A calibration curve of the Cr chemical state was constructed using XANES spectra of Cr(VI)/Cr(III) salt mixtures (Fig. 3). The intensity of the pre-edge peak intensifies with increasing Cr(VI) content. There is a near linear relationship between the pre-edge peak height and  % Cr(VI)/Cr_total_. Fig. 4a and c show the pre-edge features of normalized Cr K-edge XANES spectra in both nitrate and sulfate systems. To facilitate the comparison, the  % Cr(VI) fraction in sorption samples was estimated using the XANES calibration curve. It is important to note that “% Cr(VI) on the surface” in Fig. 4 is different from the results of macroscopic data shown in Fig. 1, which presents the “% Cr removed” from the aqueous system. The following discussion is organized based on the type of background electrolyte.

In the sulfate media (Fig. 4a, b), the amount of Cr(VI) on the magnetite surface was dependent on ionic strength under respective pH values. At basic pH values, low (0.0016 M) sulfate facilitated a very small amount of Cr(VI) (~0.1 %) on the surface [i.e., ~100 % of surface Cr is Cr(III)]. Cr(III)(OH)_3_(s) surface species are likely dominant at alkaline conditions. At acidic pH values, low sulfate yielded a large amount of Cr(VI) (60.4 %). Chromate was strongly adsorbed on Fe octahedral sites of the magnetite structure. As previously discussed, the shared charge value of sulfate (1.5) is smaller than that of nitrate (1.67), suggesting the greater affinity of sulfate on metal oxide surfaces. Its strong sorption mechanism has been well documented in different iron oxyhydroxide minerals. In goethite, CrO_4_^2−^ sorption occurs via inner-sphere complexation at near neutral pH values [33–36]. The potential for any background oxyanion to force the formation of a Cr(VI) inner-sphere surface complex could also facilitate the reduction of Cr(VI) at the magnetite surface. Considering the positively charged magnetite surfaces at acidic pH, chromate anions should be strongly attracted. However, high (0.016 M) sulfate at acidic pH resulted in low Cr(VI) (1.7 ± 0.2 %) on the surface. Competitive sulfate adsorption in the high sulfate condition might be responsible for this pH dependent Cr removal from solution (Fig. 1).

In the nitrate media (Fig. 4c, d), there was a similar ionic strength dependency in the Cr(VI) surface speciation. At acidic pH, the fraction of Cr(VI) increased from ~16 to 91.4 % with decreasing ionic strength. High [nitrate] possibly competes with the chromate anion, resulting in less Cr(VI) sorption under the high nitrate condition. At basic pH values, there was an opposite trend. Surface Cr(VI) increased from ~6.1 to ~30 % with increasing ionic strength. At alkaline pH, total Cr removal was not strongly affected by changes in ionic strength. Therefore, the increase in the Cr(VI) fraction on the surface is not attributed to an increase in the quantity of Cr(VI) adsorption. The changes in the ratio of Cr(VI)/Cr(III) is likely due phase transformation at the surface. As discussed earlier, nitrate could oxidize Fe(II) as long as a substantial quantity of nitrate is present. However, low nitrate does not effectively oxidize Fe(II), leaving some reduction capacity of magnetite. This might be the reason why more Cr(III) remained on the magnetite surface.

### Effects of pH on Cr surface speciation

When  % Cr(VI) on the surface is compared at low and high pH values under the same ionic strength, there is a much larger difference in the two electrolyte systems at low pH values.

In the low sulfate media, % Cr(VI) increased from ~0.5 to ~60 % with decreasing pH (Fig. 4b). At acidic pH, pH dependent chromate adsorption was controlling the surface speciation at low sulfate concentration. It should be noted that, under acidic pH conditions, once reduction of the Cr(VI)O_4_^2−^ has occurred, Cr(III) cations might then be desorbed from the positively charged magnetite surface since it does not readily undergo a hydrolysis reaction to form Cr(III)(OH)_3_. This could account for the lower retention of Cr(III) on the surface at low pH values.

In the high sulfate media,  % Cr(VI) increased from ~1 to ~22 % with increasing pH (Fig. 4b). At alkaline pH, auto oxidation of Fe(II) in magnetite is known to occur [12]. Newly formed Fe(III) oxyhydroxide facilitates the adsorption of Cr(VI). For Cr(VI) anions to be adsorbed at basic pH, the Cr(VI) anion must undergo a ligand exchange reaction (i.e., inner-sphere) because of negatively charged mineral surfaces. For this reason, the effect of ionic strength of specific ligands (e.g., sulfate) on Cr(VI) adsorption occurs to a much lesser extent compared to those at low pH. This supports the macroscopic observation in Fig. 1a. Because of diminished ligand effects at high pH, the Cr(VI) sorption is more susceptible to changes in other physicochemical factors such as auto-oxidation and or the activity of OH^−^, which will induce the hydrolysis reaction of Cr(III).

In the nitrate medium, a similar pH effect is observed. However, the changes are even larger. In the low nitrate system, an incomplete reduction of Cr(VI) was observed. The amount of Cr(VI) retained was as high as ~80 % in the low nitrate media at low pH. The observation of incomplete reduction of Cr(VI) in low [NO_3_] agrees with previous reports [4, 37]. Since low nitrate does not compete for the chromate adsorption, chromate anions are readily adsorbed on the surface. In the high nitrate media, however, % Cr(VI) increased from ~15 to ~28 % with increasing pH (Fig. 4d). Similar to the high sulfate system, auto oxidation of Fe(II) in magnetite is expected at high pH. Newly formed Fe(III) oxyhydroxide facilitated the adsorption of Cr(VI).

### Kinetic effects on Cr surface speciation

**Fig. 5:**
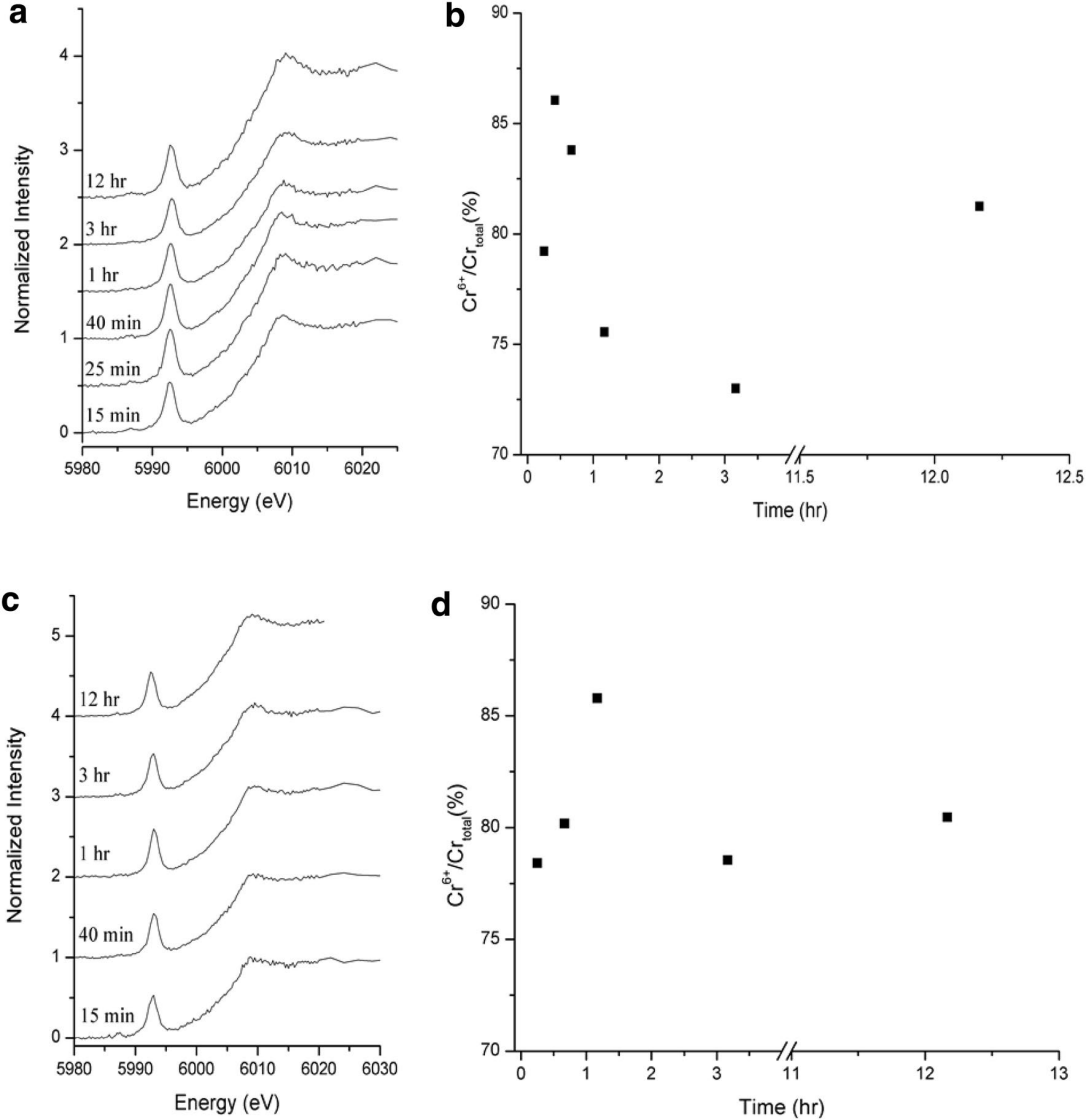
Cr surface speciation via pre-edge features of Cr K-edge XANES analysis of Cr(VI) sorption kinetic samples. **a** 0.016 M Na_2_SO_4_ at pH 4.01 ± 0.10. **b** Changes in Cr surface speciation from data assessed by XANES pre-edge analysis shown in Fig. 5a. **c** 0.01 M NaNO_3_ at pH 4.01 ± 0.10. **d** Changes in Cr speciation from data assessed by XANES pre-edge analysis shown in Fig. 5c

In the XANES analysis of equilibrium samples, it is clear that more Cr(III) is distributed in magnetite at alkaline pH in both nitrate and sulfate media (Fig. 4b, d). In other words, basic pH induced the hydrolysis of Cr(III) immediately after the Cr(VI) reduction. Several spectroscopic (e.g., X-ray photoelectron spectroscopy and X-ray absorption spectroscopy) studies reported the formation of Cr(OH)_3_, Cr(III)OOH_(s)_ and or Cr(III) bearing iron oxyhydroxide phases in the Cr(VI) reacted magnetite surfaces at pH 5–8 [15, 38, 39]. While these studies showed the Cr surface speciation in equilibrium based sorption experiments, they do not provide temporal scale information about the Cr(VI) reduction steps at the mineral–water interface. To assess the electron transfer reactions, time-resolved XANES measurements were conducted on kinetic samples at less than 12 h. We chose samples at pH 4 at low ionic strength in nitrate and sulfate media because these conditions yielded high Cr loading levels that allow us to evaluate the changes in Cr valence state during the short XANES experiments. Figure 5a and c show the pre-edge features of Cr XANES spectra in kinetic samples. Changes in % Cr(VI) on the mineral surfaces are summarized in Fig. 5b, d. During the first 12 h of sorption experiments, approximately 78–86 % of total Cr on the surfaces was still Cr(VI) in both samples. Although there are some fluctuations in the data, it is clear that only ~20 % of total Cr on the surface was Cr(III).

The  % Cr(VI) fraction on the surface increases from 15 to 30 min, suggesting that Cr(VI) adsorption was still occurring at the surfaces. Shortly after 30 min, the amount of surface sorbed Cr(VI) decreases up to ~3 h. This suggests the reduction of Cr(VI) occurred. Interestingly, between 3 and 12 h, there was an increase in the Cr(VI) fraction to ~80 %. Our interpretation is as follows. Cr(VI) reduction occurred up to ~3 h, and then electron transfer reaction was suppressed due to the formation of Cr(III) hydroxide surface precipitates. While the formation of a Cr(III)(OH)_3_(s) passivation layer can be one of explanations for the inhibitory mechanism [8–11], the transformation of adsorbent can also be pointed out at this low pH. During the Cr(VI) reduction, the depletion of Fe(II) from magnetite structure is simultaneously occurring. Both XRD and TEM analyses showed the transformation of magnetite to goethite and or maghemite (γ-Fe_2_O_3_) and then hematite (α-Fe_2_O_3_) under oxidized conditions [40]. A structural polymorph of hematite is the most common weathering product of magnetite in oxic environments. The formation of a passivation layer can also be facilitated by sulfate promoted Fe(II) dissolution. Sulfate ions could also complex with Fe(II), and increase the dissolution of Fe(II) from the magnetite structure [7, 41]. Depending on the reaction pH, the dissolved Fe(II) can precipitate as Fe(OH)_2_, and eventually oxidize to form a ferrihydrite/goethite passivation layer.

### EXAFS Analysis of steady state samples

**Fig. 6:**
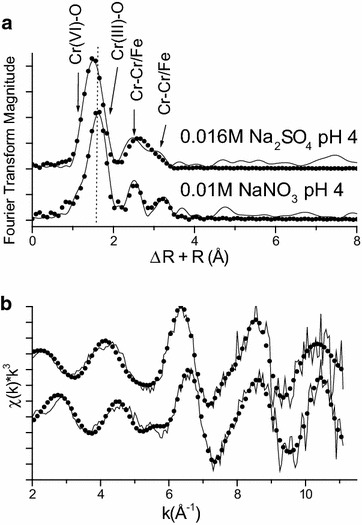
**a** Non-linear least-squares fit of normalized k^3^-weighted EXAFS spectra of Cr(VI) reacted magnetite after 48 h. A *vertical dotted line* is aligned at the peak of first shell of a bottom spectrum. **b** Corresponding Fourier transformed radial structural function (uncorrected for phase shift of backscattering atom) of EXAFS spectra. *Solid lines* and *filled black circles* represent normalized raw data and fit, respectively

Additional EXAFS analyses were conducted on sorption samples after 42 h (Fig. 6). The results are summarized in Fig. 6 and Table 1. Based on the fraction fit of oxygen shells, ~54(±7) % of total Cr on the surface was Cr(VI) in the low sulfate system at pH 4 whereas the amount of Cr(VI) was slightly lower in the low nitrate system, 49(±7) %. The difference can be seen in the position of first shell in radial structural functions (Fig. 6a). The position of the vertical dotted line is aligned at the peak of first shell in the nitrate sample. The peak position of first shell in the sulfate system is slightly lower that in the nitrate system, supporting the result of Cr valence analysis. Second and third shell features at ~3.0 and 3.5 Å were successfully fit with either Cr or Fe because of similar photo electronic scattering properties of these elements. The distance can be interpreted as a mixture of adsorbed Cr(VI)O_4_, Cr(III)O_6_ and or co-precipitated Cr(III)O_6_.

**Table 1:** Least square analysis of Cr K-edge XAS spectra

Sample		Cr(VI)–O	Cr(III)–O	Cr–Cr/Fe	Cr–Cr/Fe	% Cr(VI)	% Cr(III)	R-factor
pH 4 (± 0.02)	CN	4^a^	6^a^	1.3 (3)	0.4 (2)			
0.016 M	R	1.60 (1)	1.99 (1)	3.03 (2)	3.44 (3)	54 (±7)	46 (±7)	0.017
Na_2_SO_4_	σ^2^	0.005 (2)	0.003 (1)	0.007^a^	0.002 (1)			
pH 4 (± 0.02)	CN	4^a^	6^a^	1.2 (1)	0.6 (3)			
0.01 M	R	1.65 (1)	1.98 (1)	3.00 (3)	3.48 (2)	49 (±7)	51 (±7)	0.030
NaNO_3_	σ^2^	0.008 (4)	0.016 (1)	0.007^a^	0.002 (1)			

Estimated errors for CN: ±20 % and R: ±0.01Å [12]

*CN* Coordination number, *R* inter atomic distances (Å), *σ* ^*2*^ Debye–Waller factor (Å^2^)

^a^ Fixed parameters for the first Cr–Cr/Fe shell is according to the study [12]

Based on the first shell analysis, it is clear that both Cr(VI)O_4_ and Cr(III)O_6_ surface species are present. Coordination number of Cr–Cr/Fe distance at 2.91 Å is about one. This corresponds to the edge sharing mononuclear Cr(VI)O_4_ on FeO_6_ in goethite [38]. Edge sharing mononuclear Cr(III) on FeO_6_ in goethite [42] can be also considered along with Cr(III) co-precipitates [43]. A distance (2.98 Å), which is consistent with edge of two MeO_6_ polyhedral with Me as Fe and or Cr, was reported in the XAS analysis of Cr(III) surface precipitates (γ-CrOOH) in ferrihydrite at pH 4 [43]. Feff/XRD generated Cr–Cr interatomic distance in bracewellite, CrO(OH), at 2.97 Å [44].

There is an additional Cr–Cr/Fe shell at ~3.5 Å in both samples. The similar distance was previously reported as multinuclear Cr surface species on the hematite (0001) surface via grazing incident-XAFS analysis [45]. Double corner sharing of CrO_6_ to Cr/FeO_6_ yields in a similar distance [42]. Feff/XRD simulation of bracewellite structure shows the Cr–Cr interatomic distance of ~3.4 Å. The Cr–Fe distance of 3.4 Å was reported in Cr(III) substituted α-FeOOH [48].

## Conclusions

Magnetite readily removed dissolved Cr(VI) from solution in the presence of nitrate and sulfate. Like other oxyanions, sorption of Cr(VI) increases with decreasing pH. While the effects of ionic strength were more pronounced in sulfate media than nitrate media, total Cr retention was greater in sulfate solutions than in nitrate solutions. The oxidation of Fe(II) (as surface bound or crystal defects) in magnetite by nitrate suppressed Cr(VI) reduction, resulting in less Cr removal in nitrate media. In sulfate media, competitive adsorption of sulfate was more of an important factor at acidic pH. XANES analysis revealed that the Cr(VI) surface reduction occurred at high pH in low ionic strength (0.01 M) of both electrolytes. However, such electron transfer reactions were suppressed at low pH, resulting in more adsorbed Cr(VI) on the surfaces. Because of the structural alternation of adsorbent (i.e., formation of passivation layers such as Cr(III) precipitates and Fe(III) oxyhydroxides), the kinetics of Cr(VI) reduction was slow after 3 h as evident in the XANES and EXAFS analysis. Adsorbed Cr(VI) surface species dominated during the initial several hrs, and nearly 50 % of total Cr on the surface was adsorbed Cr(VI) anions in both sulfate and nitrate media. This suggests that adsorption processes of Cr(VI) anions on magnetite surfaces is as important as the reductive precipitation of Cr(III) in explaining the removal of Cr(VI) with magnetite. In assessing the stability of sorbed Cr in magnetite, it might be important to consider the desorption process of Cr(VI) anions with respect to common ions in natural waters.

## Experimental

### Materials

Synthetic magnetite (Fe_3_O_4_) nanopowder was obtained from Nanostructured and Amorphous Materials, Inc. (Houston, TX) Particle size was 50–100 nm with >99 % purity. The following ACS grade chemicals were prepared in degassed ultrapure water (18.2 MΩ): sodium nitrate, sodium sulfate, nitric acid, sulfuric acid, and sodium hydroxide. Solutions of ACS grade sodium chromate tetrahydrate, sodium acetate, and 3-propanesulfonic acid (MOPSO) were prepared in 0.01 M and 0.1 M NaNO_3_ and 0.0016 M and 0.016 M Na_2_SO_4_. These concentrations represent 0.01 and 0.1 ionic strengths for the background solutions, respectively. Standards were prepared using the NIST traceable ICP-MS standard ammonium dichromate (1000 mg/L Cr in 3 % nitric acid, 99 %, Ricca Chemical Company). The ACS grade chemicals potassium chromate [Cr(VI)] and chromium oxide [Cr(III)] were used for the X-ray energy calibration.

### PZSE determination

Potentiometric acid–base titrations were conducted using 0.2 g/L magnetite nanopowder in 0.01, 0.05 and 0.1 M NaNO_3_ backgrounds. Solutions were prepared in individual 50 mL Nalgene polypropylene sterile high-performance (PS) centrifuge tubes. The nanopowder was hydrated for 24 h on an end-over-end shaker at 20 rpm; afterwards a predetermined amount of 0.01–0.1 M NaOH and HNO_3_ were added to each tube resulting in an approximate pH range of 3–10. The samples were then shaken on an end-over shaker for 24 h. The final pH values were plotted against total acid concentration and the point of intersection in the batch titration curves was used to find the point of zero salt effect (PZSE) [46].

### Batch sorption experiments

Magnetite suspensions were prepared in 5 g/L solid to solution ratio in 50 mL PS high-performance centrifuge tubes. Samples were hydrated in 0.001, 0.1 M NaNO_3_, 0.0016 or 0.016 M Na_2_SO_4_ degassed background solutions for 10 h on an end-over-end shaker at 30 rpm. It is important to note that no magnetic stir bar was used due to the magnetic property of magnetite. pH values were adjusted before and after hydration using 0.01–1 M NaOH and either H_2_SO_4_ or HNO_3_ depending on the background electrolyte. Samples were prepared over an approximate pH range of 3–12; no buffer was used. The solutions were spiked with 0.5 mM Na_2_CrO_4_∙4H_2_O and returned to the shaker for 24 h. After the experiment the pH of each sample was recorded and aliquots were collected, filtered through a 0.2 µm polyvinylidene fluoride (PVDF) filter. Filtrates were diluted with 1 % Suprapur^®^ nitric acid, and then analyzed using an inductively coupled plasma mass spectrometer (ICP-MS) Thermo Scientific X Series 2 that was calibrated using a Cr(VI) NIST traceable ICP-MS standard. The Cr(VI) was assured using a spectrophotometric method [47].

### XAS analysis

All XAS samples were prepared at room temperature. Kinetic samples were freshly prepared at the Stanford Synchrotron Radiation Laboratory (SSRL), Menlo Park, CA. For equilibrium samples, sorption samples were prepared at 10 g/L for collection and analysis purposes. The hydration, spiking, and aliquot collection procedure were identical to the sorption experiments. Kinetic samples for X-ray absorption near edge structure (XANES) analysis were prepared in 50 mL high-performance centrifuge tubes with 5 g/L solid to solution ratio with either 0.01 M NaNO_3_ or 0.016 M Na_2_SO_4_ and acidic pH (4.01 ± 0.10). Acidic pH was chosen because of this reaction condition provides sufficient Cr loading level for rapid (i.e., single scan) XANES measurements. Sodium acetate (50 mM) was used as a buffer solution. Tubes were placed on an end-over-end shaker at 30 rpm and one tube was sacrificed for each time interval. Mineral suspensions were spiked with 1 mM Na_2_CrO_4_∙4H_2_O and sampled at 15, 25, 40 min, and 1, 3, 12 h. Each sample was filtered using vacuum filtration on PVDF filter papers, trapped between Kapton tape, and immediately analyzed at the beamtime. We chose the room temperature analysis with one scan over the cryo measurements since the sample loading time in a cryo cell and holder requires more than 5 min. For the extended X-ray absorption fine structure spectroscopy (EXAFS) measurement, equilibrium samples after 48 h were used.

All samples were analyzed at beam line 4–3 at SSRL. The electron storage ring was operated at 3 Ge V energy with a current range of 80–100 mA. The energy calibration was performed at 5989 e V using the first derivative of a Cr foil XANES spectrum. Fluorescence-yield Cr K-edge spectra were collected using a 4 element vortex detector. The monochromator was a Si(111) double-crystal with a non-fixed exit slit. Sample holders were oriented at 45° to the unfocused incident beam. All samples were run at room temperature.

To assess the change in Cr valence state at the mineral–water interface during the sorption reaction, a Cr pre-edge peak standard curve was constructed using the Cr K-edge pre-edge peak of Cr(VI)/Cr(III) mixtures. All reference spectra were collected in transmission mode. The reference salts, K_2_Cr(VI)O_4_ and Cr_2_(III)O_3_, were mixed to give a range (0–100 %) of Cr(VI) concentration, ground with a diamonite mortar and pestle, and the fine powder was trapped in Kapton tape.

The data reduction of bulk XANES spectra was performed using the SixPACK/IFEFFIT interface [48]. Because of fast sorption reactions, only one spectrum was collected per kinetic sample. The following data normalization was carried out at approximately 5800–6150 eV. A Gaussian function was used for normalization of the pre-edge region and a quadratic function was used for the post-edge region. Extended X-ray absorption fine structure spectroscopy analysis was conducted in two equilibrium sorption samples after 42 h according to the method described in Arai and Livi [49]. Only two spectra were averaged and splined up to 11.1 Å^−1^. Because of only two scans, any noise in Fourier transformed radial structural function (RSF) feature >3.6 Å was unable to be fit. The structural refinement data of chromite and K_2_CrO_4_ were used to generate single scattering paths for Cr(III)–O, Cr(VI)–O, Cr(III)–O and Cr–Fe [50, 51]. In order to assess the fraction of Cr(III) and Cr(VI), a sum of each fraction was set to unity, and each fraction was multiply to CN of each Cr path. Based on tetrahedral structure of Cr(VI) and octahedral structure of Cr(III), CN was fixed at 4 and 6, respectively. The rest of fitting parameters were floated unless otherwise mentioned in the text.
